# Cognitive control impairment in ax‐continuous performance test in patients with schizophrenia: A pilot EEG study

**DOI:** 10.1002/brb3.3276

**Published:** 2023-10-10

**Authors:** Bing Li, Chao‐meng Liu, Li‐na Wang, Wen‐qing Jin, Wei‐gang Pan, Wen Wang, Yan‐ping Ren, Xin Ma, Yi‐lang Tang

**Affiliations:** ^1^ Hebei Provincial Mental Health Center Baoding China; ^2^ Hebei Key Laboratory of Major Mental and Behavioral Disorders Baoding China; ^3^ The Sixth Clinical Medical College of Hebei University Baoding China; ^4^ The National Clinical Research Center for Mental Disorders & Beijing Key Laboratory of Mental Disorders, Beijing Anding Hospital Capital Medical University Beijing China; ^5^ Advanced Innovation Center for Human Brain Protection Capital Medical University Beijing China; ^6^ Department of Psychiatry and Behavioral Sciences Emory University School of Medicine Atlanta Georgia USA; ^7^ Mental Health Service Line Atlanta VA Medical Center Decatur Georgia USA

**Keywords:** cognitive control, electroencephalography, proactive control, reactive control, schizophrenia

## Abstract

**Objectives:**

This study aimed to investigate the mechanism of cognitive control impairment in patients with schizophrenia (SPs) using electroencephalogram (EEG).

**Methods:**

A total of 17 SPs and 17 healthy controls (HCs) were included in this study. We measured the EEG activity, whereas they performed the AX‐continuous performance test which consisted of the preparatory phase and the response phase. The MATRICS Consensus Cognitive Battery (MCCB) was used for cognitive function, and the Positive and Negative Syndrome Scale (PANSS) was used for clinical symptom assessment. A univariate linear regression model was used to explore the relationships among behavioral index, event‐related potentials (ERPs), rhythmic oscillation power, and score of MCCB and PANSS.

**Results:**

A significant difference was found in response accuracy and reaction time (RT) during the preparatory phase between patients and HCs (*p* < .05). During the response phase, the SPs exhibited longer RT than the HCs (*p* < .05). Analysis of the ERPs revealed that the amplitude of P3a on BX clues was significantly smaller in SPs than in HCs (*p* < .05). Additionally, the midline frontal theta power of neural oscillation was significantly lower in the SPs than in NCs both during the preparatory and response phases. The accuracies on BX clues (*r* = .694, *p* = .002) and d'context (*r* = .698, *p* = .002) were positively correlated with MCCB scores.

**Conclusion:**

The present study revealed that patients with schizophrenia have deficits both in proactive and reactive cognitive control, with a greater reliance on reactive control during conflict resolution. The neural mechanisms of the cognitive control impairment may involve the inability to engage additional neural resources for proactive control, and a reduction in frontal midline theta power during both proactive and reactive control. The severity of proactive control impairment is positively correlated with an increased tendency to rely on reactive control.

## Graphical abstract

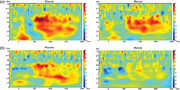

## 1 INTRODUCTION

Schizophrenia is a severe mental illness with a lifetime prevalence rate of approximately 0.6% in China (Huang et al., 2019). A meta‐analysis estimated that the pooled median point and 12‐month prevalence of psychotic disorders worldwide are 3.9 and 4.0 per 1000 persons, respectively (Moreno‐Küstner et al., 2018). In addition to positive and negative symptoms, cognitive impairment is considered a core feature of schizophrenia, with substantial implications for treatment and prognosis (Green et al., 2019). Patients with schizophrenia (SPs) often exhibit numerous cognitive impairments, including perceptual, nonsocial, and social cognitive processes. There may be a mechanism underlying these cognitive domain defects, namely, the function to actively represent context information in working memory and focus on information relevant to the current task to guide behavior (Barch & Ceaser, 2012). Braver (2012) proposed the framework of the “dual mechanisms of cognitive control,” including two distinguishable mechanisms of cognitive control. The proactive mechanism of control, also known as the early selection and maintenance of goal‐relevant information in anticipation of a challenging event, serves to ideally guide attention. In contrast, the reactive mechanism of control involves the stimulus or event‐driven activation of goal‐relevant information, without prior anticipation or preparation for processing (Braver et al., 2021). Specifically, proactive control is driven by cue information, allowing for advanced predictions and strategies for upcoming conflicts via maintaining the representation of task‐related cue information before the probe stimulus emerges. On the other hand, reactive control is driven by probe information and involves resolving conflicts based on task‐related information immediately after the probe stimulus occurs (Braver et al., 2009).

Context information relates to task goals that appear in advance, including task instructions, previous stimulus processing results, and target information, which are maintained in working memory and can bias one's attention and guide behavior. The impairment in context information processing can explain schizophrenia's defects in working memory, episodic memory, executive function, attention, inhibition, and language processing (Cohen et al., 1999). Therefore, exploring the mechanism of cognitive control impairment may help uncover the essential causes of cognitive impairment in schizophrenia, guide the development of effective interventions, and improve patient outcomes.

In studies investigating cognitive control deficits in schizophrenia using event‐related potentials (ERPs), paradigms involving conflict detection and monitoring are commonly employed. Existing studies have often focused on components of the P3 family, which has been extensively researched in the studies of schizophrenia (Turetsky et al., 2007). The P3 component reflects attention processing and comprises an early component P3a and a late component P3b. Kropotov et al. (2019) showed that P3 amplitude did not alter in conflict detection but decreased in response inhibition in SPs. In a study by Fallgatter and Müller (2001), no differences were initially observed between chronic SPs and healthy controls (HCs) regarding the amplitude and latency of P3 in a Go/No Go task. However, upon expanding the sample, it was revealed that the amplitude of No Go P3 in SPs was lower compared to HCs (Fallgatter & Müller, 2001; Fallgatter et al., 2003). Furthermore, Ertekin et al. (2017) discovered that the amplitude of P3 in the central and parietal cortex of SPs was significantly lower in the No Go trials than in the Go trials. However, in the frontal region, the amplitude of P3 decreased similarly in the No Go and Go trials (Ertekin et al., 2017).

Various rhythmic neural oscillations are involved in cognitive control, including theta and gamma band oscillations. During cognitive control processing, frontal theta power is significantly enhanced, reflecting conflict and control processing (Cavanagh & Frank, 2014). The coordinated activity between regions in the cognitive control networks, with the dorsolateral prefrontal cortex playing an important role (Howard et al., 2003), is essential in cognitive control. The AX continuous performance test (AX‐CPT) paradigm is an enhanced iteration of the CPT, and it is well known for its ability to distinguish between active control and reactive control. It has become a seminal approach for assessing cognitive control proficiency (Chun et al., 2018). There exist several comparable tasks, including probabilistic reversal learning, operation and symmetry span, preparing to overcome prepotency (POP), and various Stroop tasks. However, these tasks have been criticized for their lack of construct validity, their measurement of distinct facets of executive control, their limited usage in schizophrenia research, or their inability to distinguish specific cognitive impairments from generalized deficits (Barch et al., 2009; Carter et al., 2012). In light of these concerns, the current study has chosen to employ the AX‐CPT paradigm, primarily due to its commendable performance as acknowledged by the Cognitive Neuroscience Treatment Research to Improve Cognition in Schizophrenia committee (Chun et al., 2018). In a study using the AX‐CPT, researchers found that SPs have lower frontal midline theta power during proactive control compared to the HCs, suggesting impairment in proactive control (Ryman et al., 2018). Another investigation employing the POP task to assess cognitive control in SPs revealed that frontal theta power did not show significant reduction, whereas gamma power did, irrespective of medication usage. This finding implies that the impairment of gamma power may underlie the deficiency in cognitive control observed in schizophrenia (Minzenberg et al., 2010). The potential discrepancy between the two outcomes could potentially be attributed to the utilization of distinct task paradigms. Most previous studies have focused on specific aspects of cognitive control procession, failing to provide a comprehensive analysis and discussion of behavior, ERPs, and rhythmic oscillations in local brain regions throughout the entire cognitive process. The lack of a holistic approach to studying the dynamic processes in cognitive control may bias the conclusions. To address this gap, our study aims to comprehensively examine the behavior, ERPs, and rhythmic oscillations in regional brain activity during both the preparatory and response phases of cognitive control. Our goal is to gain a clear understanding of cognitive control impairment in SPs. We hypothesized that: (a) The cognitive control of SPs is impaired, mainly in proactive control; (b) there will be alterations in ERP components, theta, and gamma power between cortices of SPs during the preparatory and response phases of cognitive *control; and (c) these alterations are correlated with the patient's cognitive function and clinical symptoms*.

## 2 MATERIALS AND METHODS

### 2.1 Participants

Twenty‐one clinically stable SPs were recruited from Beijing Anding Hospital, Capital Medical University, and 32 age‐, gender‐, and education‐matched HCs with normal hearing and right‐handed were enrolled. Inclusion criteria for both groups were as follows: (a) age between 18 and 60; (b) the education level junior high school or above. Both groups were evaluated by two experienced psychiatrists to estimate whether they met the diagnostic criteria of DSM‐IV. (c) Symptom stabilization criteria: Antipsychotic treatment regimen has not changed in the last 3 months. Exclusion criteria for both groups were as follows: (a) any neurological illness; (b) apparent sensation and movement disorders that make it impossible to use a computer; (c) metal implants in the brain; (d) pregnancy; (e) any other diagnosis of mental disorders; and (f) any current psychiatric or neurological diagnosis or treatment for controls. The study was approved by the ethical committee of Beijing Anding Hospital. Written informed consent was obtained from all participants.

### 2.2 Clinical and cognitive assessment

Clinical data were collected from patients using the Positive and Negative Syndrome Scale (PANSS). The PANSS scale comprises a total of 30 items, which are primarily categorized into 7 positive scales, 7 negative scales, and 16 general pathological scales. Additionally, there are three supplementary items designed to assess the risk of attack. Each item is assessed on a 7‐point scale ranging from 1 to 7, with well‐defined project specifications and grading criteria. A higher cumulative score on the PANSS scale indicates more pronounced symptom severity. The evaluation of the PANSS scale is conducted by psychiatrists who have received training to ensure consistency in their assessments (Fong et al., 2015). Cognitive data were collected using the Chinese version of the Measurement and Treatment Research to Improve Cognition in Schizophrenia (MATRICS) Consensus Cognitive Battery (MCCB) (Shi et al., 2015) both for patients and HCs. The MCCB has chosen 10 subtests out of a pool of over 90 tests, which encompass 7 cognitive domains: working memory, word learning, visual learning, social cognition, information processing speed, reasoning and problem‐solving, and attention/alertness. The MCCB places particular emphasis on the essential attributes that cognitive tests employed in clinical trials should possess, including high retest reliability, reusability, correlation with functional status, sensitivity to drug response, practicality, and patient tolerance (Plichta et al., 2023).

### 2.3 Multisensory AX‐CPT task

**FIGURE 1:**
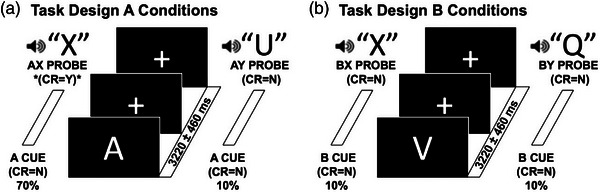
Multisensory AX‐continuous performance test (AX‐CPT) task: Participants were instructed to put their left index finger on the button “Q” (which represents “yes”) and the right index finger on the button “P” (which represents “no”) on keyboard and pressed the button as soon as possible when the probe stimulus is heard. There are four kinds of stimuli pairs: AX, AY, BX, and BY. As shown on the left panel A, the correct response (CR) was “yes” (Y) when the letter X followed A (target sequence AX; 70% of trials). The three remaining stimuli pairs (AY in panel A, BX and BY in panel B) all required a “no” (N) response and each occurred on 10% of trials. Letter sequences were presented in a pseudorandom order. The total number of trials collected was as follows: 280 AX trials and 40 of each of the remaining trial types. The interstimulus interval was 3220 ms jittered by 460 ms. The intertrial interval was 4520 ms jittered by 460 ms.

E‐prime 3.0 software (Science Plus Group) was used for stimulus presentation and recording of behavioral data. In the AX‐CPT Task (Chun et al., 2018; Ryman et al., 2018), participants were presented with a series of visual cue stimuli—letters A, R, V, P, S, and E (duration: 500 ms)—and auditory probe stimuli—letters X, Q, F, I, M, and U (duration: 500 ms), and instructed to respond “yes” when the letter X followed the letter A or respond “no” at other conditions. All cue stimuli that were not A were referred to as “B cues,” and all probe stimuli that were not X were subsequently referred to as “Y probes.” AX stimulus pair accounted for 70% (280 trials). AY, BX, and BY stimulus pairs accounted for 10% (every 40 trials). The interstimulus interval was 3220 ms, jittered by 460 ms, and the intertrial interval was 4520 ms, jittered by 460 ms (Figure 1). Before the electroencephalogram (EEG) assessment, participants received instructions and completed at least a 10‐trail practice until their performance indicated an understanding of the task. Participants needed to achieve at least 70% accuracy in the practice before EEG recording.

Confirmatory factor analysis of the AX‐CPT revealed that BX trials primarily involved context information processing during the preparatory phase, whereas AY trials primarily involved response preparation during the response phase (MacDonald et al., 2005). Context information processing is the core process of proactive control, whereas response preparation reflects the primary process of reactive control. Thus, BX trials mainly reflect a proactive control function, whereas AY trials mainly reflect a reactive control function. Previous studies did not divide the preparatory phase from the response phase, which were different. Previous studies did not differentiate between the preparatory and response phases, despite their evident differences. In the cue phase, one should prepare for the probe according to A or B, the so‐called preparatory phase. In the probe phase, one should react to X or Y in the context of the previous cue, the so‐called response phase.

The d'context index is derived from the difference between the accuracy rate on AX and the error rate on BX, serving as a measure of proactive control. A lower d'context score indicates a diminished capacity for integrating contextual information. Additionally, a low d'context score signifies impaired proactive control, leading individuals to rely more on reactive control in conflict situations.

### 2.4 Electrophysiological data recording and processing

Electrophysiological data were collected on a Brain Products EEG system utilizing a 64‐electrode EEG cap and a sampling rate of 2500 Hz with BrainVision Recorder software, in a shielded room with active electrodes, with no observed spectral peaks at 50 Hz (*DC power for the EEG device*). *The reference electrode was FCz*, the grounding electrode was AFz, and the impedance between the scalp and the electrode was less than 5 kΩ. The participants were instructed to keep their heads as still as possible during the experiment. The participants’ EEG data were recorded while performing the AX‐CPT task.

The data underwent standard preprocessing steps (Cavanagh et al., 2009) using MATLAB (MathWorks; The MathWorks, Inc.) and EEGLAB (http://sccn.ucsd.edu/eeglab/) (Delorme & Makeig, 2004). Concerning data preprocessing, the date of the FC*z* electrode was restored and then re‐referenced the electrode to the whole brain average reference electrode. The filter was set to 0.05–100 Hz band‐pass filtering. As the components of ERP still needed to be analyzed, the current data were saved and backed up for time–frequency analysis, and then 30 Hz low‐pass filtering was applied. The onset of A and B and probe stimulus of stimuli pair AX, AY, and BX were used as the reference time points, respectively, and the −200 ms period before was used as the baseline for correction. All EEG data were browsed, checked, and removed segments with artifacts; insufficient electrode data were interpolated, and only trials with correct responses were kept.

EEG data were analyzed and processed using the toolbox EEGLAB 13.0.0b based on MATLAB 2013b (MathWorks). According to the previous literature (Jung et al., 2000), the remaining physiological artifacts were discarded using the Infomax Independent Component Analysis algorithm. The ERP data were segmented by a time window ranging from 200 ms before to 2000 ms after stimulation. Then, data were down‐sampled to 500 Hz. As in previous publications (Ryman et al., 2018), time–frequency measures were calculated by multiplying the fast Fourier‐transformed power spectrum of the single trial data with the fast Fourier‐transformed power spectrum of a set of complex Morlet wavelets. Power was extracted from these large windows and converted to decibel (dB) scale based on the average precue activity from −300 to −200 ms. *Theta (4–7 Hz) power of FCz that represents midline region were examined*. The selection of the time window for gamma oscillation was informed by prior research, encompassing a substantial duration spanning from 500 to 1000 ms (Redick & Engle, 2011). Given the distinction between the visual nature of the AX‐CPT task cue stimulus employed in this investigation and the auditory nature of the detection stimulus, along with the dissimilar activation times of these two stimulus responses, the theta oscillation time window subsequent to visual stimulation was determined to be 250–450 ms, whereas the theta oscillation time window following auditory stimulation was determined to be 125–325 ms (Turetsky et al., 2007).

### 2.5 Statistics analysis

SPSS21.0 software was used for statistical data analysis. The continuous variables with normal distribution were expressed by mean ± standard deviation among the indexes. Paired *t*‐test, two‐factor mixed analysis of variance (ANOVA) of group condition was used for various comparisons. Post hoc test was used for further analysis. Continuous variables that did not have a normal distribution were represented by percentiles P50 (P25, P75), and the Wilcoxon signed‐rank test was used. Categorical data were expressed by constituent ratio, and the McNemar test was used. The univariate linear regression model was used to analyze the correlation among behavioral index, ERP, rhythmic oscillation power, and PANSS and MCCB scores. The test level was set at *α* = .05, two tail.

## 3 RESULTS

### 3.1 Demographics and clinical data

A total of 21 SPs and 32 HCs were enrolled. We used the binomial distribution with two response options to determine the exclusion thresholds based on accuracy, which are 55% for AX cue‐probe trials and 65% for all other trials. Two cases were removed due to performance below the exclusion threshold, and another two patients were excluded due to poor EEG data quality in the SPs group. In HCs group, 5 cases were removed due to performance below the exclusion threshold, and another 10 due to poor EEG data quality. Finally, the analysis included 17 SPs and 17 HCs. SPs had lower MCCB total scores than HCs. There were no significant differences in age, sex, marital status, and education level between the two groups (Table 1).

**TABLE 1:** Comparison of demographics and cognitive characteristics between patients with schizophrenia (SPs) and healthy controls (HCs).

Characteristic	SPs (*n* = 17)	HCs (*n* = 17)	*t/x* ^2^ Value	*p* Value
Age (years)	33.76 ± 13.20	34.00 ± 12.72	0.054	.958
Sex (male/female)	11/6	11/6	–	–
Marital status (married/other)	5/12	6/11	1.134	.714
Education level (years)	14.29 ± 3.06	14.53 ± 2.98	0.259	.799
MCCB score	45.35 ± 10.44	51.82 ± 6.67	2.187	.044
Illness duration (years)	12.36 ± 12.83	–	–	–
PANSS positive scale	12.53 ± 4.09	–	–	–
PANSS negative scale	18.06 ± 4.25	–	–	–
PANSS general psychopathology	28.53 ± 7.27	–	–	–
PANSS total score	59.12 ± 13.70	–	–	–

Abbreviations: MATRICS, Measurement and Treatment Research to Improve Cognition in Schizophrenia; MCCB, MATRICS Consensus Cognitive Battery; PANSS, Positive and Negative Syndrome Scale.

### 3.2 Behavioral data

**FIGURE 2:**
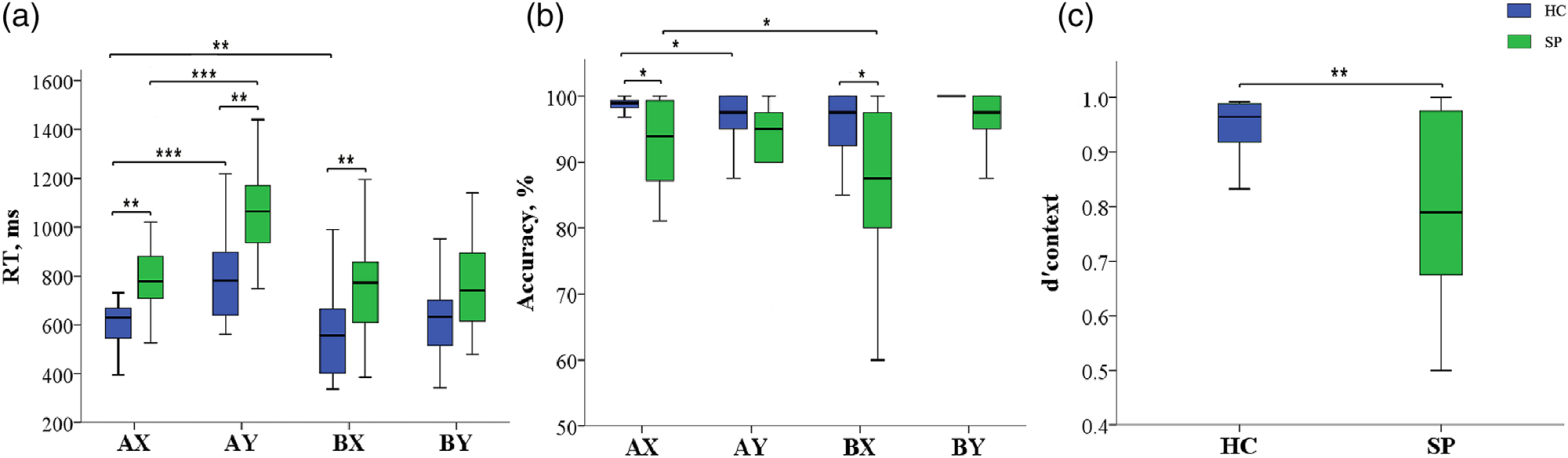
(a) Mean reaction time (RT) for each group and condition. (b) Mean accuracy for each group and condition. (c) D'context for healthy controls (HC) and patients with schizophrenia (SPs) group. Error bars represent standard errors of the means. Asterisks indicate significant difference between groups and conditions (*p < .05, **p < .01,***p < .001).

For the reaction time (RT) of the two groups (Figure 2a), the groups (SP vs. HC) × condition (AX vs. AY and AX vs. BX, respectively) two‐factor mixed‐measures ANOVAs revealed significant differences in the main effects of condition (AX vs. AY: *F* = 127.449, *p* < .001; AX vs. BX: *F* = 5.535, *p* = .025) and group (AX vs. AY: *F* = 8.362, *p* = .007; AX vs. BX: *F* = 6.361, *p* = .017), but no significant difference in group × condition interaction (AX vs. AY: *F* = 2.473, *p* = .126; AX vs. BX: *F* = .786, *p* = .382). The paired *t*‐test revealed that when the two groups were compared on AY, BX, and AX, the patient group's RT was significantly longer than the control group's (*t* = −3.595, *p* = .002; *t* = −3.348, *p* = .004; *t* = −3.005, *p* = .008). According to the within‐group comparison, the RT was significantly longer on AY than on AX (*t* = −7.918, *p* < .001) while significantly shorter on BX than AX (*t* = 3.242, *p* = .005) in the HC group. The RT was also significantly longer on AY than on AX (*t* = −8.147, *p* < .001), but no significant difference between BX and AX (*t* = .846, *p* = .410) in the SPs group.

The accuracy comparison between the HCs and SPs groups revealed that the patient group accuracy was significantly lower than the control group on AX (*Z* = −2.353, *p* = .019) and BX (*Z* = −2.525, *p* = .012). The within‐group comparison results indicated that the accuracy was significantly lower on AY than on AX (*Z* = −2.062, *p* = .039) in HCs, whereas the accuracy was significantly lower on BX than on AX (*t* = 2.349, *p* = .032) in SPs (Figure 2b). Paired *t*‐test displayed that the d'context of SPs (0.80 ± 0.16) was significantly lower (*t* = 3.574, *p* = .003) than that of HCs (0.94 ± 0.06) (Figure 2c).

### 3.3 ERPs results

According to previous studies and ERP waveforms, the grand average P3b amplitudes between 400 and 600 ms at CP1, CP2, CP*z*, P1, P2, and P*z* channels were selected in the preparatory phase of cognitive control to explore the ERPs of superior parietal cortex during cognitive control. The grand average P3a amplitudes between 220 and 330 ms at FC*z*, C1, C2, C*z*, CP, CP2, and CP*z* channels were selected in the response phase to explore the ERPs of the parietal cortex during cognitive control.

**FIGURE 3:**
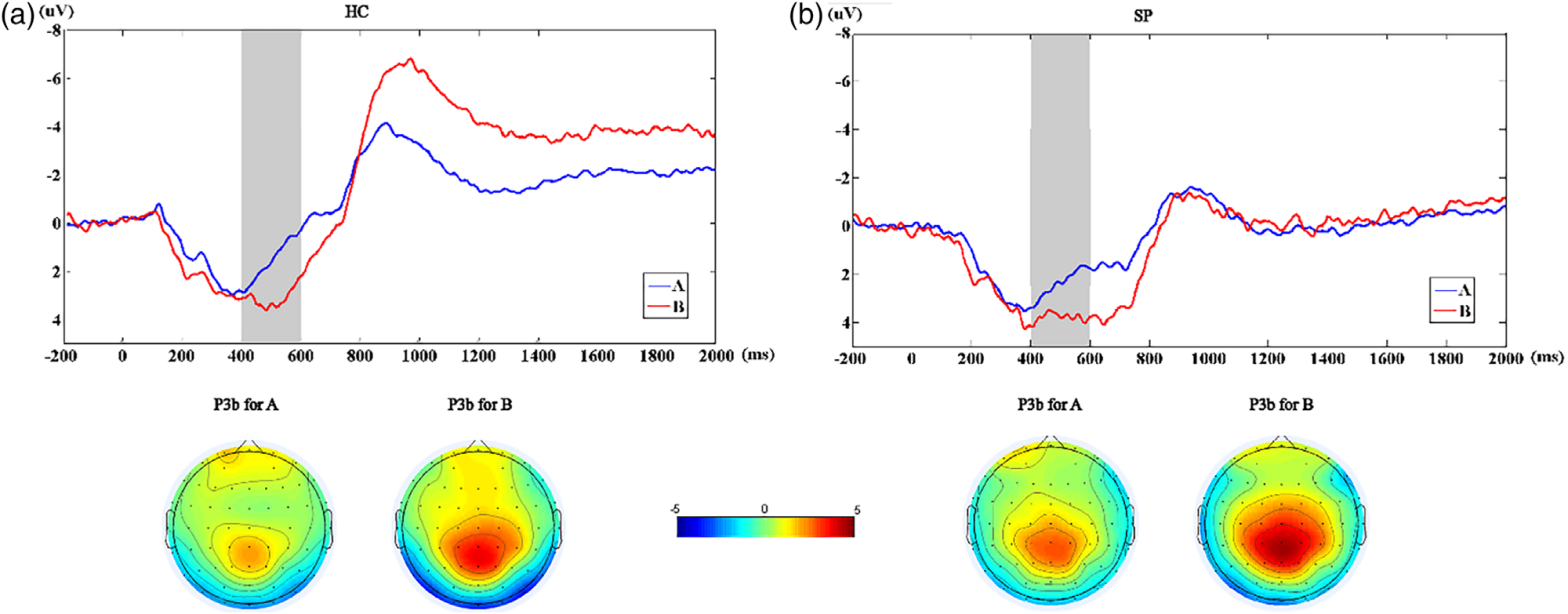
(a) The grand‐average event‐related potentials (ERPs) of superior parietal cortex (SPC) in healthy controls (HC) group on cues A and B (top panel). The gray area shows the time windows (400–600 ms) of P3b waveforms. The topographical maps assessed between 400 and 600 ms following cue onset on cues A and B (bottom panel). (b) Same as (a) in patients with schizophrenia (SPs) group.

Concerning P3b, the group (HCs vs. SPs) × condition (A vs. B) two‐factor mixed‐measures ANOVA on P3b amplitude revealed a main effect of cue (*F* = 19.852, *p* < .001). Follow‐up tests indicated that P3b amplitudes were larger on cue B (HCs: 3.14 ± 2.27 μV; SPs: 3.79 ± 2.83 μV) than on cue A (HCs: 1.57 ± 1.70 μV; *t* = −4.545, *p* = .001; SPs: 2.40 ± 2.11 μV; *t* = −2.522, *p* = .023) for both groups (see Figure 3 and Supplementary material 1).

**FIGURE 4:**
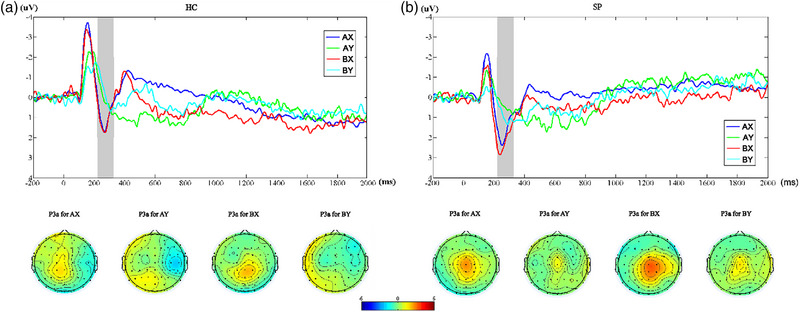
(a) The grand‐average event‐related potentials (ERPs) of parietal cortex in healthy controls (HC) group on probe AX, AY, BX, and BY (top panel). The gray area shows the time windows (220– 330 ms) of P3a waveforms. The topographical maps assessed between 220 and 330 ms following cue onset on all probes (bottom panel). (b) Same as (a) in patients with schizophrenia (SPs) group.

The mean amplitudes of P3a were significantly higher in HCs than in SPs (*t* = 2.343, *p* = .032) on BX. According to the within‐group comparisons, the mean amplitudes of P3a were significantly higher on BX than on AX (*t* = −2.339, *p* = .033), and those on AY were significantly lower than on AX (*Z* = −3.627, *p* < .001) in HCs. The mean amplitudes of P3a were also significantly lower on AY than on AX (*Z* = −3.243, *p* = .001) in SPs, but those on BX have no significant difference than on AX (see Figure 4 and Supplementary material 2).

### 3.4 Neural oscillations power results

**FIGURE 5:**
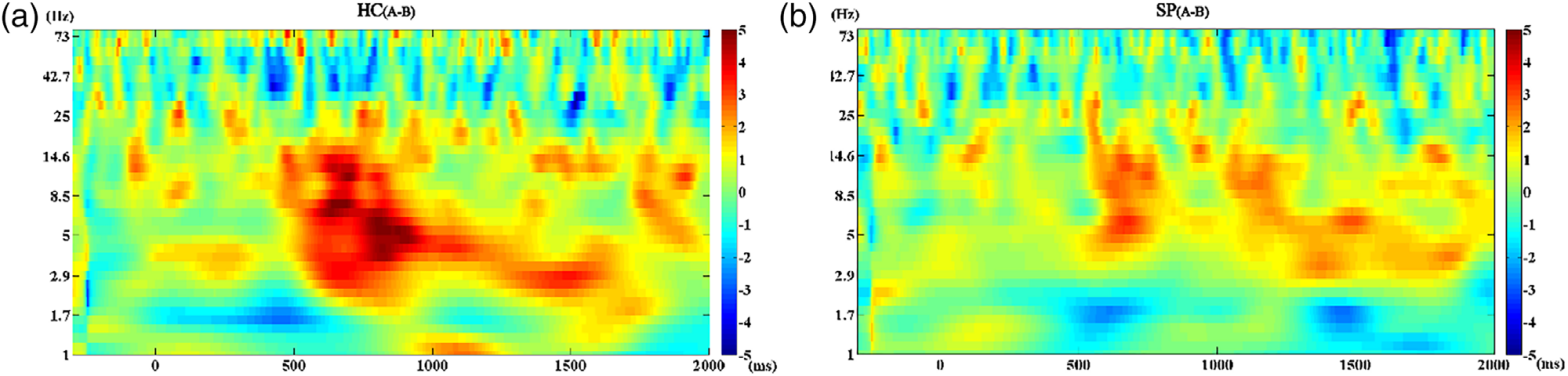
(a) Results of comparisons (A vs. B) of theta power across all frequencies in healthy controls (HCs), which indicated no differences after FDR correction. (b) Same as (a) in patients with schizophrenia (SPs).

ANOVA (A vs. B) on the frontal midline theta power in the time window of interest revealed a significant group effect (*F* = 9.367, *p* = .004) during the preparatory phase. According to the between‐group comparisons, the theta power of SPs was lower than that of HCs on cue A (*t* = 2.696, *p* = .016) and B (*t* = 3.421, *p* = .004). The within‐group comparisons did not exhibit significant differences (Figure 5 and Supplementary material 3).

**FIGURE 6:**
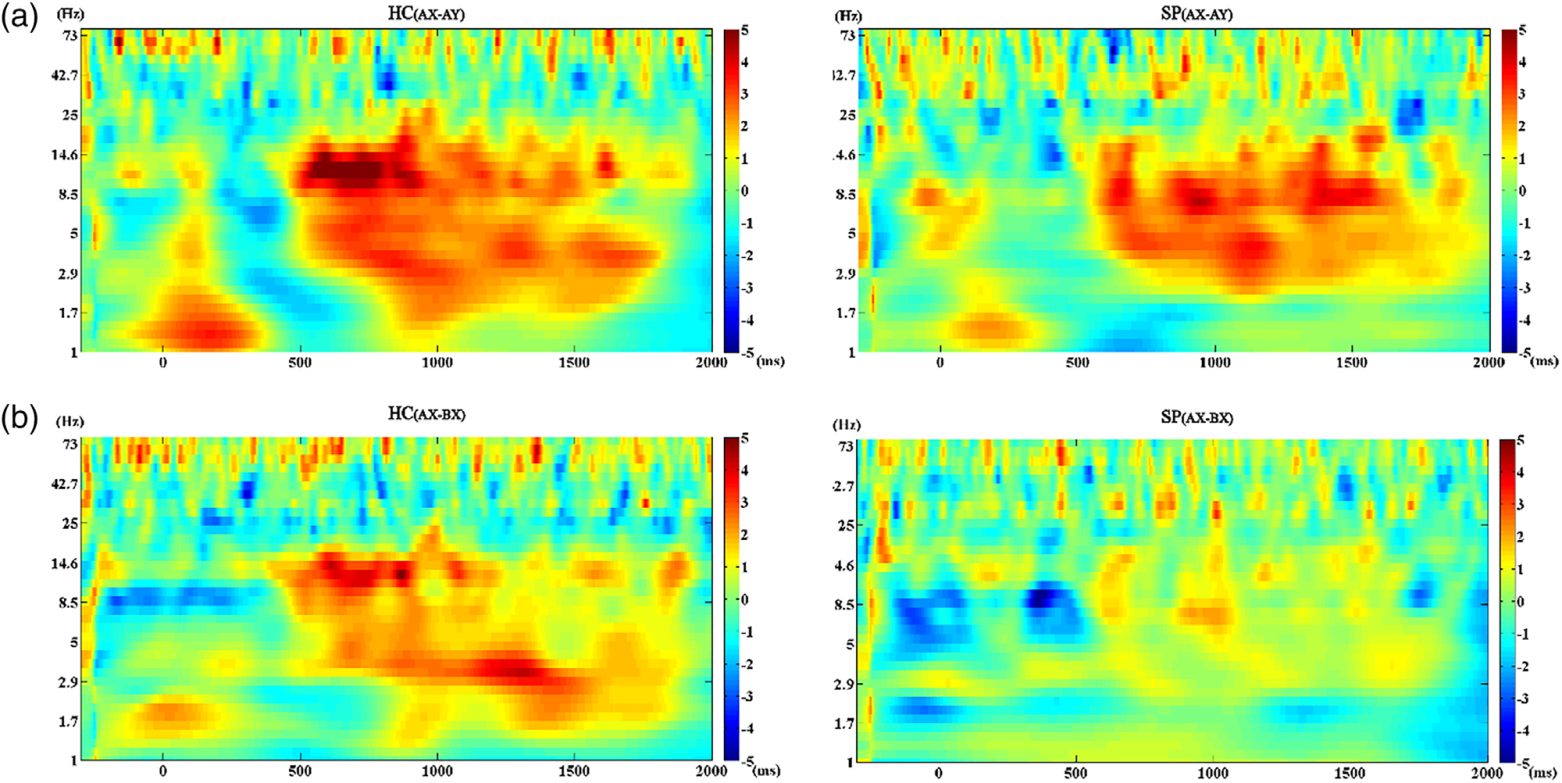
(a) Results of comparisons (AX vs. AY) of theta power across all frequencies in healthy controls (HCs) (left panel) and patients with schizophrenia (SPs) (right panel), which indicated no differences after FDR correction. (b) Results of comparisons (AX vs. BX) of theta power across all frequencies in HCs (left panel) and SPs (right panel), which indicated no differences after FDR correction.

ANOVA (AX vs. AY) revealed a significant main effect of group (*F* = 38.951, *p* < .001) and condition (*F* = 4.758, *p* = .037) on the frontal midline theta power in the time window of interest during the response phase. ANOVA (AX vs. BX) revealed a significant main effect of the group (*F* = 34.183, *p* < .001) on the frontal midline theta power in the time window of interest. Further analyses disclosed that FC*z* frontal midline theta power of SPs was lower than that of HCs on either AX (*t* = 6.775, *p* < .001), AY (*t* = 4.925, *p* < .001), or BX (*t* = 5.662, *p* < .001) probe. The within‐group comparisons reveal no significant differences (Figure 6 and Supplementary material 4).

### 3.5 Correlation analysis

**FIGURE 7:**
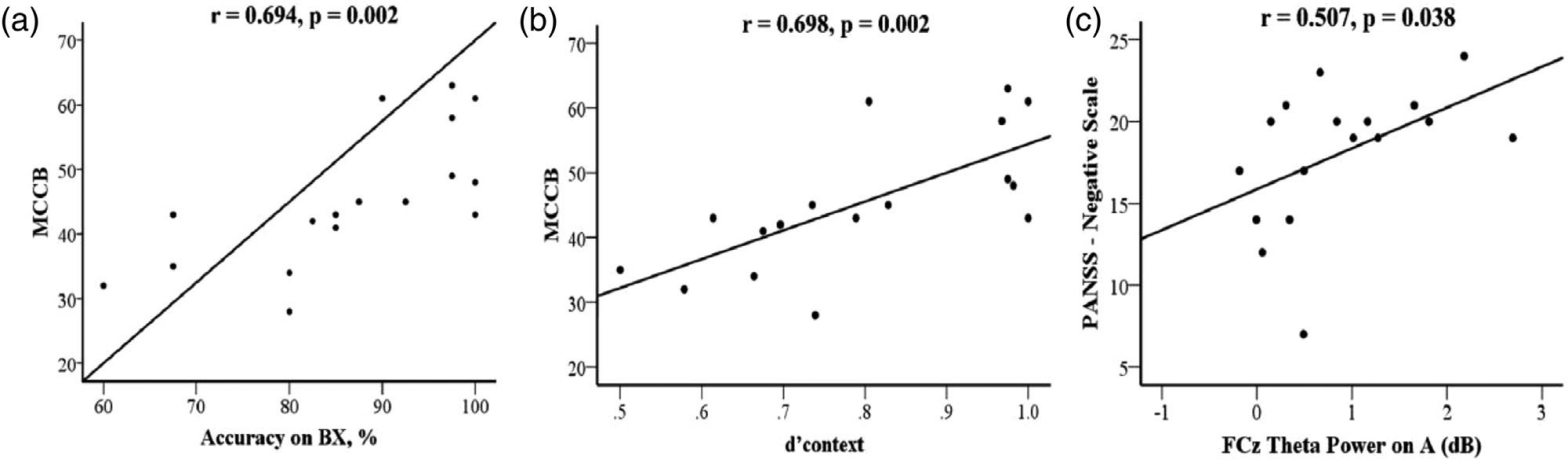
In patients with schizophrenia (SPs), (a) the accuracy on BX was positively correlated with MATRICS Consensus Cognitive Battery (MCCB) score; (b) d'context was positively correlated with MCCB score; (c) FC*z* theta power on A was positively correlated with Positive and Negative Syndrome Scale (PANSS)‐negative scale score. MATRICS, Measurement and Treatment Research to Improve Cognition in Schizophrenia.

According to the previous results, we also explored the correlations between the response accuracy on BX, RT on AY/BX, d'context, mean P3a amplitudes on BX, FC*z* theta power on A/B/AY/BX, and scores of MCCB, PANSS in SPs. The results displayed that the accuracy on BX was positively correlated with MCCB score (*r* = .694, *p* = .002); d'context was positively correlated with MCCB score (*r* = .698, *p* = .002); FC*z* theta power on A was positively correlated with PANSS‐negative scale score (*r* = .507, *p* = .038) (Figure 7).

## 4 DISCUSSION

This study examined the behavior, ERPs, and neural oscillations power associated with cognitive control using the AX‐CPT paradigm in SPs. Moreover, our study is the first to distinguish the preparatory phase and the response phase. The findings revealed impairments in both proactive and reactive control in SPs, with P3 amplitudes, and frontal midline theta power showing correlations with cognitive function and negative symptoms.

Analysis of behavioral data analysis showed significant decrease in response accuracy and slower RT for SPs compared to HCs on BX trials. The lower response accuracy and longer RT were associated with poorer cognitive function. The results reflected a significant decline in proactive control in SPs. In AY trials, there were no significant differences in response accuracy between the two groups, but SPs exhibited significantly longer RT than HCs, indicating significant impairment in reactive control. Although SPs could still maintain the ability to make a correct response, their efficiency was significantly decreased. When facing conflicts, SPs tended to rely more on reactive control. Additionally, our study showed that the d'context scores of SPs were significantly lower than HCs, which is consistent with previous studies (Niendam et al., 2018; Smucny et al., 2018). Based on current evidence, it is evident that reactive control of SPs is also significantly impaired, resulting in decreased executive efficiency and slower RT. However, the ability to make a correct response is not significantly reduced, which may be because SPs tend to use reactive control when they encounter conflicts; therefore, the function is partially maintained.

During the preparatory phase of cognitive control, we observed that the amplitude of P3b was significantly greater on the B condition than on the A condition in both SPs and HCs. This suggests that more neural resources were allocated to proactive control than to reactive control. This finding can be attributed to the requirement of keeping the cue in mind when watching B, which involves inhibiting the prepotent response upon the probe's appearance. In contrast, when watching A, individuals simply wait to react to the probe.

During the response phase, the amplitude of P3a on BX was significantly smaller in SPs than in HCs. This finding, along with the lower accuracy of SPs on BX, suggests that SPs could not recruit sufficient neural resources for proactive control to inhibit the prepotent response. Notably, HCs exhibited a significantly larger P3a amplitude on BX trials compared to AX trials, whereas no difference was observed between the two conditions in SPs. This suggests that inhibiting the prepotent response requires more neural resources which SPs fail to achieve.

Our results are in line with a previous study that examined P3a component alterations during conflict resolution using the Go/No Go task and found a smaller P3a component in SPs than in HCs (Chun et al., 2013). Additionally, a recent study using the AX‐CPT task to investigate the modulation of P3 during the cognitive control process in HCs (Xu et al., 2020) revealed a significantly higher P3b amplitude during proactive control compared to reactive control, after cue presentation. However, previous studies have often overlooked the distinction between the preparatory phase and the response phase. In contrast, our study highlights the specific ERP alterations in each phase, complementing previous findings. Overall, our findings are consistent with previous research but provide novel insights by examining the features of ERP alterations in the preparatory and response phases separately.

We demonstrated that SPs exhibited lower midline frontal theta power than that in HCs during both the preparatory and response phases, suggesting potential neural basis for the defects in proactive and reactive control observed in SPs. Activation of frontal midline and its connection with the lateral prefrontal cortex have been implicated in cognitive control processing (Cavanagh & Frank, 2014). Current evidence related to cognitive control has primarily focused on the activity of theta and gamma band oscillations. Theta band oscillations are known to reflect error detection and correction process (Trujillo & Allen, 2007). Ryman et al. (2018) conducted a study utilizing the AX‐CPT task to compare the rhythmic oscillations during the cognitive control process between SPs and HCs, and they found that SPs did not exhibit the same enhancement of frontal midline theta power as HCs on the B cue, suggesting impaired proactive control. They also observed a general decrease in theta power in SPs, which may reflect a common impairment in the cognitive control process of schizophrenia. However, they did not observe impairment in gamma oscillation in SPs. Contrary to the above findings, a previous study using the POP task (Minzenberg et al., 2010) reported no significant impairment in theta power in SPs, regardless of medication usage. Our results contribute new evidence by highlighting impaired theta oscillations in SPs during cognitive control. In addition, previous research did not identify differences between SPs and HCs in the preparatory phase. The lower theta power observed in SPs in the preparatory phase aligns with observed defects in proactive control, consistent with the ERP alteration found in our study.

We found that accuracies on BX and the d'context score were positively correlated with the total score of MCCB in SPs, suggesting a positive correlation between proactive control and cognition in SPs. Additionally, the frontal midline theta power during the preparatory phase of reactive control of SPs was positively correlated with the PANSS‐negative scale score, suggesting that when the negative symptoms were more severe, SPs are more inclined to reactive control which more neural resources are allocated to. Previous studies have suggested that cognitive impairment was closely related to negative symptoms in schizophrenia, with similar occurrence, progression, and outcome characteristics, and may share the same pathological basis (Harvey, 2012). However, some argue that both are characteristic lesions of schizophrenia and distinct clinical syndromes (Yuan et al., 2016). Our findings support the former point of view, as we found the more severe the impairment of proactive control, the more likely SPs were to use reactive control, and the worse the overall cognitive function. This suggests that cognitive impairment and negative symptoms are closely related to cognitive control. Several limitations to the current study need to be acknowledged. First, the sample size included was relatively small. Second, the subjects were prone to fatigue and could not always remain motionless, due to the long duration of the AX‐CPT task and the monotonous nature of the operation. As a result, we had to exclude several subjects due to the noise of EEG data. Third, most patients were medicated (including antipsychotics); therefore, the effects of medications cannot be ruled out. Finally, this is a case–control study, and we did not conduct follow‐up, so we cannot reflect the longitudinal changes in cognitive control and determine whether the differences are causal to illness.

Our findings first distinguished the preparatory phase from the response phase, revealing deficits in both proactive and reactive control in SPs. We also found that SPs rely more heavily on reactive control during conflict resolution. The neural mechanisms that contribute to the cognitive control impairment may involve the inability to engage additional neural resources for proactive control during the response phase, as well as a reduction in frontal midline theta power during both proactive and reactive controls. The severity of proactive control impairment is positively correlated with an increased tendency to rely on reactive control, which in turn is associated with a decline in overall cognitive function. It is important to acknowledge that the conclusions drawn from this study should be further substantiated through the implementation of large sample and multicenter studies, given the limited size of the current sample and different disease stages and their potential impact on reliability and generalizability. By understanding the neurophysiological mechanism of cognitive control impairments in schizophrenia, our findings may help future research aiming to develop targeted interventions that can enhance cognitive functioning treatment outcomes of SP patients.

## AUTHOR CONTRIBUTIONS

Bing Li, Yan‐ping Ren and Xin Ma were involved in the study design. Bing Li, Chao‐meng Liu and Li‐na Wang performed the experiments. Chao‐meng Liu and Wei‐gang Pan contributed to the analysis and interpretation of data. Wen‐qing Jin, Wen Wang and Yi‐lang Tang wrote the manuscript. All the authors revised critically and approved the final version of the manuscript and agree to be accountable for all aspects of the work in ensuring that questions related to the accuracy or integrity of any part of the work are appropriately investigated and resolved. All persons designated as authors qualify for authorship, and all those who qualify for authorship are listed.

## CONFLICT OF INTEREST STATEMENT

The authors declare no conflicts of interest.

### PEER REVIEW

The peer review history for this article is available at https://publons.com/publon/10.1002/brb3.3276.

## Supporting information

Supporting information

## Data Availability

The dataset generated during this study is available from the corresponding author upon reasonable request.
