# Supplementary material for: Cognitive control impairment in ax‐continuous performance test in patients with schizophrenia: A pilot EEG study
Source: Brain Behav. 2023 Oct 10;13(12):e3276. doi: 10.1002/brb3.3276 (PMC10726902; doi:10.1002/brb3.3276)

**Supplementary material 1.** Mean amplitudes of P3b between 400 and 600 ms after cue onset for each group at each cue. Asterisks indicate significant difference between groups or conditions (*p<0.05, **p<0.01).

**
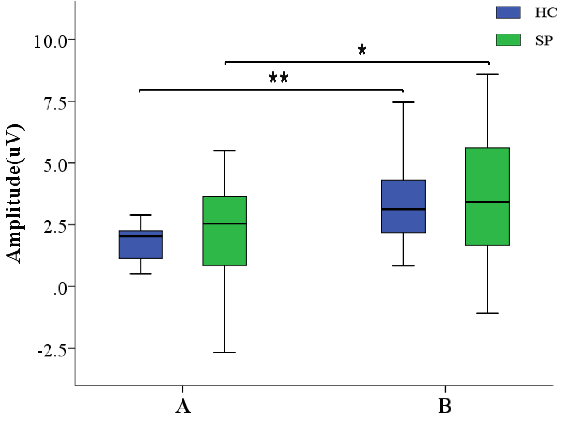
**

**Supplementary material 2.** Mean amplitudes of P3a between 220 and 330ms after probe onset for each group at each probe. Asterisks indicate significant difference between groups or conditions (*p<0.05, **p<0.01, ***p<0.001).


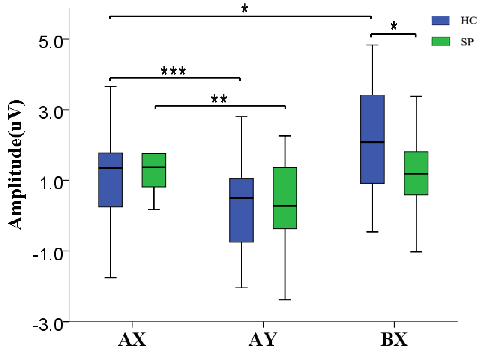


**Supplementary material 3.** Mean FCz theta power in the time window of interest for each group at each cue. Asterisks indicate significant difference between groups (*p<0.05, **p<0.01).


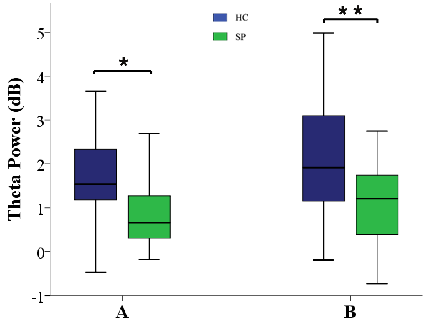


**Supplementary material 4.** Mean FCz theta power in the time window of interest for each group at probe AX, AY and BX. Asterisks indicate significant difference between groups (***p<0.001).


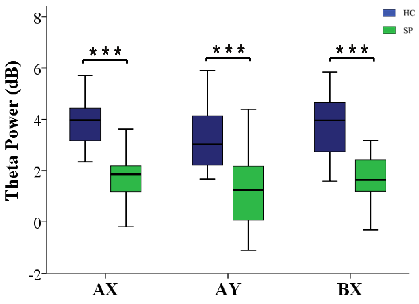

Supplement: Supplementary file 1 — Supporting information [file BRB3-13-e3276-s001.docx]
